# Scene Recognition Using Deep Softpool Capsule Network Based on Residual Diverse Branch Block

**DOI:** 10.3390/s21165575

**Published:** 2021-08-19

**Authors:** Chunyuan Wang, Yang Wu, Yihan Wang, Yiping Chen

**Affiliations:** 1 School of Electronic and Electrical Engineering, Shanghai University of Engineering Science, Shanghai 201620, China; wangcy@sues.edu.cn (C.W.); chenyiping@sues.edu.cn (Y.C.); 2 Shanghai Institute of Satellite Engineering, Shanghai 200240, China; wuyywu566@163.com

**Keywords:** scene recognition, squeeze and excitation, diverse branch block, capsule network, residual convolution

## Abstract

With the improvement of the quality and resolution of remote sensing (RS) images, scene recognition tasks have played an important role in the RS community. However, due to the special bird’s eye view image acquisition mode of imaging sensors, it is still challenging to construct a discriminate representation of diverse and complex scenes to improve RS image recognition performance. Capsule networks that can learn the spatial relationship between the features in an image has a good image classification performance. However, the original capsule network is not suitable for images with a complex background. To address the above issues, this paper proposes a novel end-to-end capsule network termed DS-CapsNet, in which a new multi-scale feature enhancement module and a new Caps-SoftPool method are advanced by aggregating the advantageous attributes of the residual convolution architecture, Diverse Branch Block (DBB), Squeeze and Excitation (SE) block, and the Caps-SoftPool method. By using the residual DBB, multiscale features can be extracted and fused to recover a semantic strong feature representation. By adopting SE, the informative features are emphasized, and the less salient features are weakened. The new Caps-SoftPool method can reduce the number of parameters that are needed in order to prevent an over-fitting problem. The novel DS-CapsNet achieves a competitive and promising performance for RS image recognition by using high-quality and robust capsule representation. The extensive experiments on two challenging datasets, AID and NWPU-RESISC45, demonstrate the robustness and superiority of the proposed DS-CapsNet in scene recognition tasks.

## 1. Introduction

With the high-speed improvement of the performance of optical remote sensors, it is very economical and effective to obtain high-resolution remote sensing (RS) images [1]. Therefore, it is very important to understand its semantic information effectively for their extensive and deep applications in land planning, urban change, traffic control, disaster monitoring, military, agriculture, and forestry [2,3]. Each RS image scene is automatically assigned a specific semantic label according to its content, which is called RS image scene recognition, and this is one of the hottest topics in the field of RS image processing [4,5].

**Figure 1:**
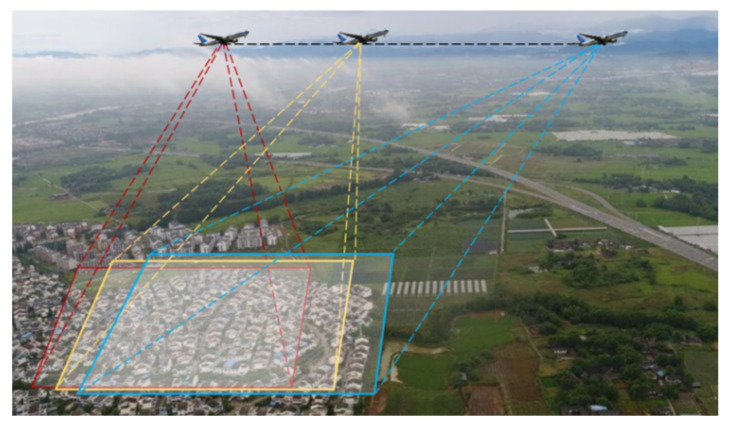
Aerial view to retrieve RS images.

**Figure 2:**
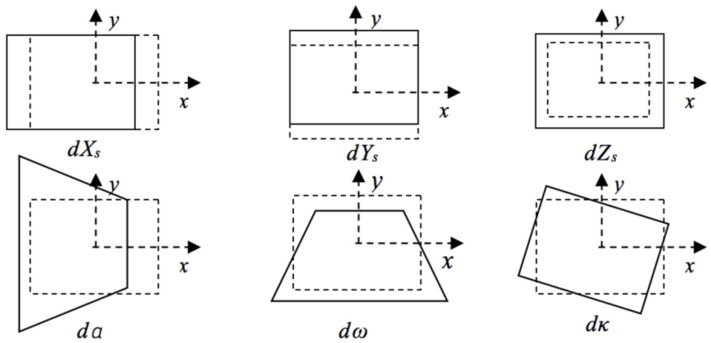
Image deformation caused by changes in the external orientation elements.

**Figure 3:**
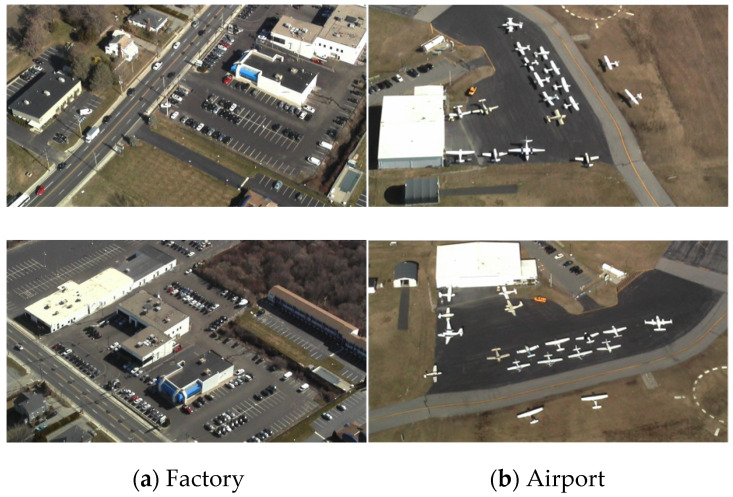
Examples for multiview RS images.

However, due to the special bird’s eye view image acquisition mode, as shown in Figure 1, RS images have different degrees of affine transformation and scale changes [6], which make traditional methods ineffective. Figure 2 shows the image deformation caused by the change of the external orientation elements of the imaging sensor, in which the change of location *X Y Z* and the azimuth angle *K* produce linear deformations, such as translation, scale, and rotation deformations, while changing the yaw angle α and pitch angle ω causes the nonlinear deformation of the image. For example, in Figure 3, the airport and factory images are obtained from different angles. It is visible that the objects in multiview RS images often appear with different degrees of deformations. There are significant topological changes in the shape, length, and width of objects as well as spectral and textural diversity caused by different illumination conditions.

In addition, some objects in different classes of scenes have similar shapes and textures but a different spatial arrangement, which also creates great recognition difficulties. Thus, it is still challenging to realize automatic and high-precision scene recognition from RS images [7,8]. Developing advanced and efficient techniques to further improve the efficiency and the accuracy of scene recognition has very important practical significance and an urgent demand for widespread applications [9,10].

In the past few decades, a lot of methods have been put forward for scene recognition that have mainly been based on human-defined feature extraction including local binary patterns, Gabor wavelets, the gray level co-occurrence matrix [11,12,13], etc. However, it is difficult to apply human-defined methods in practice because of the complexity and diversity of RS scenes, which promotes the development of more powerful and accurate recognition schemes. With the high-speed development of deep learning, RS image analysis tasks have made many breakthroughs in efficiency and accuracy [14,15]. Compared to conventional feature descriptors, deep learning has the advantages of end-to-end automatic mining of informative, representative, and multilevel features. Therefore, using deep learning technology has attracted great attention and efforts [16]. Owing to its advantages in data processing power and hierarchical learning abilities, CNNs (convolutional neural networks), which are one of the representatives of deep learning, have been extensively applied in information extraction and RS image recognition and have achieved many state-of-the-art results [17,18,19,20]. Although CNNs perform well in many tasks, they also have many limitations. CNNs cannot explicitly learn the relationship between feature locations, which makes scene recognition difficult when identifying similar objects in different location relationships. This means that CNN only determine whether there is an object rather than understanding the semantic relationships between all parts of the object. Moreover, CNNs use pooling operations to reduce the number of parameters and to keep translation invariance, but conventional pooling methods cause the loss of important details and location information. For a 24 × 24 size object in an RS image, only about 1 pixel remains after 4-layer pooling, which makes it difficult to be distinguished.

Recently, in order to address the limitation of CNNs in learning the relationships between features in different locations of the image, Hinton [21] advanced the concept of the “capsule”, which is an interpretable deep learning model and is considered as a powerful alternative to CNN. Different from the traditional deep learning models based on scalar neurons, the capsule network uses vector capsule representation to describe informative features. An understanding of RS image processing can be deepened by using the part–whole relationship in images. Each capsule learns equivariant representation, which is more robust to the spatial relationships between the objects and the changes of their poses in the image. Capsule networks have been extensively applied in classification, recognition, segmentation, and prediction tasks [22,23,24,25,26] due to their superior feature encoding characteristics. However, the scene of RS image has a large field of vision and high background complexity, which will produce strong interference for recognition. The original CapsNet [27] only employs two convolution layers and involves a great quantity of training parameters to attempt to explain all of the contents of an image, which are unsuitable for scene recognition with a complex background.

In this paper, a novel capsule network is proposed for scene recognition in RS images, which has been named DS-CapsNet and employs a new multi-scale feature enhancement module and a new Caps-SoftPool method by using the residual convolution architecture, Diverse Branch Block (DBB), Squeeze and Excitation block (SE), and the Caps-SoftPool. DBB enriches the feature space by using the multi-branch topology structure with different receptive fields and paths of various complexities and embeds the complicated structures into convolutional operations, which can improve performance on the premise of keeping the original inference–time overhead. Here, we construct a residual structure DBB to alleviate the gradient vanishing problem caused by increasing the depth in the deep neural network. The purpose of adding SE is to increase the input features by using channel interdependence, to enhance the ability of network to stress informative features, and to weaken the weight of less prominent features from a global perspective. Specifically, the SE block is designed to be embedded between two convolutional layers, which overcomes the shortcoming that convolution only considers the characteristic information of the local receptive field and enhances the global receiving capabilities of the network. In order to alleviate the over-fitting problem and to reduce the feature parameter number, a new capsule pooling method (Caps-SoftPool) is proposed to further improve the output quality and the robustness of capsules. The DS-CapsNet is constructed using four conv layers for extracting low-level features and a series of capsule convolutional layers for extracting high-level capsule features. The novel DS-CapsNet achieves a competitive and promising performance in RS image recognition.

The contributions of this paper are summarized below:

1. A novel end-to-end deep DS-CapsNet is structured to extract multilevel, multi-scale, and informative RS image features to provide a semantic strong capsule representation to increase scene recognition accuracy;

2. A new multi-scale feature enhancement module is designed by using the residual-structure DBB and SE, which makes full use of the attention mechanism and multi-scale information to highlight the channel-wise salient and informative features, which plays a positive role in improving the ability of feature representation;

3. A new capsule pooling method (Caps-SoftPool) between capsules is proposed to provide a high-quality and robust capsule representation that will not lose important detail information and will meanwhile lessen the number of capsules need to prevent an over-fitting problem.

4. Our novel model can achieve excellent scene recognition accuracies that are better than some state-of-the-art algorithms on two challenging datasets.

The structure of this paper is as follows: The background is illustrated in Section 2. Section 3 describes the novel architecture of the DS-CapsNet in detail. In Section 4, the effects of different factors are analyzed, and the experimental results are discussed. Finally, the conclusion is given in Section 5.

## 2. Background

In general, CNN is a forward artificial neural network, inspired by a biological vision system [28]. A deep CNN can be built by the nonlinear superposition of multiple convolution (Conv) layers and multiple pooling layers [29].

The capsule network proposed by Hinton offsets the deficiency of CNN in theory. Compared to CNN, the biggest modification is each capsule is that it outputs an activation vector instead of a scalar value. Regarding the robustness of CNN, rotation, translation, and scale is poor, which usually reduces its performance. The output of the capsules in a capsule network is a vector representing the attributes of the object. A vector formula not only allows a capsule to detect features, but also allows it to learn and recognize its invariances, thus forming a strong but lightweight feature representation pattern.

The capsule network consists of three main parts: a convolution layer (ConvL), a primary capsule layer (CapsL), and a fully connected (FC) CapsL, where the ConvL in the CapsNet is the same as it is in a CNN. The primary CapsL redistributes the input of the convolutional results into multiple capsules and includes width, height, batch size, and channel-number. Additionally, the output of the primary CapsL includes width, height, batch size, capsule number, and vector length. The following FC CapsL, which adopts dynamic routing to update the coupling coefficient and generates the recognition results, outputs 10 vector capsules at least. Each capsule consists of two parts: the orientation represents the object properties, and the length indicates the occurrence probability of the corresponding class.

For a capsule *j*, its input is the dynamically weighted sum of transformation of all the prediction values *u_j_*_|_*_i_* from capsules in the previous Conv kernel, as shown below

(1) $$s_{j}=\sum_{i}c_{ij}u_{j|i}{}_{}$$

(2) $$u_{j|i}=W_{ij}u_{j}$$

where *s_j_* is capsule *j*’s input; *c_ij_* represents a coupling coefficient dynamically determined by the prediction contribution of capsule *i* of the previous layer; *u_j_*_|*i*_ is the prediction value from capsule *i*; *W_ij_* represents the transformation matrix of the feature mapping function; and *u_i_* represents the output of the *i*-th capsule.

Furthermore, the output of a capsule is normalized from exceeding 1 by a nonlinear activation function called “squashing”. It is formulated as follows:

(3) $$v_{j}=\frac{{∥s_{j}∥}^{2}}{1+{∥s_{j}∥}^{2}}\;\frac{s_{j}}{∥s_{j}∥}$$

where *v_j_* is the normalized output of the *j*-th capsule. By operating normalization, the short vector is close to 0, and the long one is almost 1.

In the training process, the network encodes the whole image context by dynamic routing, which gradually learns the corresponding part–whole relationship to achieve a higher-level capsule.

In our network, the loss function calculated by the margin loss can be given as:

(4) $$L_{m}=T_{m}max\left(0,k^{+}-∥v_{m}{∥}^{2}\right)+\;\lambda \left(1-T_{m}\right)max{\left(0,∥v_{m}∥-k^{-}\right)}^{2}$$

when *m* class really exists, then *T_m_* = 1, and *k*^+^, *k*^−^, and λ are parameters that are stated during training. The overall loss is the sum of the marginal losses of all of the output in the final layer.

## 3. Methods

**Figure 4:**
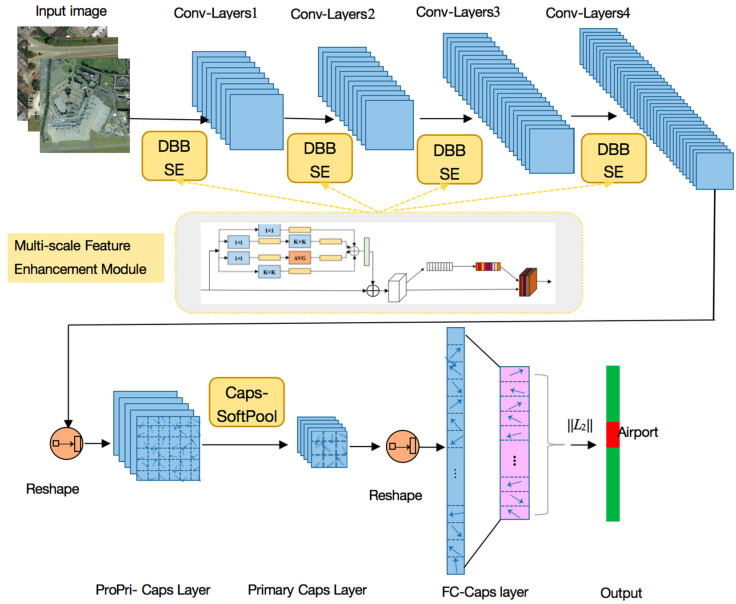
The main architecture of the DS-CapsNet.

To improve the RS scene recognition accuracy, we constructed a novel DS-CapsNet architecture in which ConvLs are deepened and strengthened by a new multi-scale feature enhancement module that employs the residual-structure Diverse Branch Block (DBB) and Squeeze and Excitation (SE) mechanism, and the CapsL is operated by a new capsule soft-pooling method between the capsules. The main architecture of DS-CapsNet is shown in Figure 4.

### 3.1. Residual Diverse Branch Block

The basic capsule network only uses two conventional ConvLs with a large-sized kernel to extract the features. In our work, four ConvLs were employed by using DBB to enhance the representational ability of single convolution. DBB combines different branches with diverse complexities and scales to enrich the feature space [30]. It has been proven that integrating two different capacities branches is better than integrating two identical high-capacity branches (e.g., a 1 × 1 conv and a 3 × 3 conv replaced two 3 × 3 convolutions), which brings enlightenment to the design of ConvsNet.

Different from the innovation of ConvsNet architecture, DBB adopts a complex “microstructure” in training while keeping the macrostructure unchanged so that it can be directly embedded into any existing architecture as a drop-in replacement. Through this “heterogeneity” in training, the obtained model can achieve higher performance with higher complexity in training and can be returned to the original structure for inference.

It is assumed that if the input channel number is *C*, the output channel number is *D*, and the Conv size is *K* × *K*, then the Conv kernel is $F\in R^{D\times C\times K\times K}$, and the optional bias parameter is $b\in R^{D}$. To facilitate subsequent merging, the bias parameter is formulated as $REP\left(b\right)\in R^{D\times H\times W}$. A convolution can be defined as follows:

(5) O=I*F+REP(b)

The value of the *j*-th output channel at (*h*, *w*) is calculated by

(6) $$O_{j,h,\omega }=\underset{c=1}{\sum }\underset{u=1}{\sum }\underset{v=1}{\sum }F_{j,c,u,v}X_{\left(c,h,\omega \right)u,v}+b_{j}$$

where $X\left(c,h,\omega \right)\in R^{K\times K}$ represents the sliding window. The linear property of convolution can be derived from the above formula, including the homogeneity and additivity,

(7) $$I*\left(pF\right)=p\left(I*F\right),\forall p\in RI*F^{\left(1\right)}+I*F^{\left(2\right)}=I*\left(F^{\left(1\right)}+F^{\left(2\right)}\right)$$

**Figure 5:**
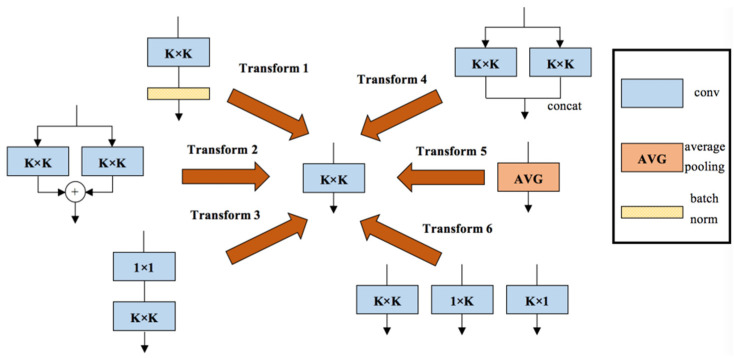
The six transformations included in DBB.

**Figure 6:**
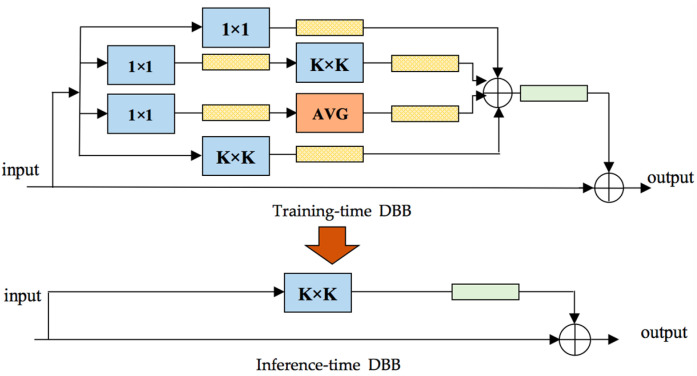
A representative example of DBB.

As shown in Figure 5, the DBB includes six transformations: (1) Conv batch normalization (BN) merging; (2) branch addition; (3) Conv sequence merging; (4) depth concatenation; (5) average pooling transformation; (6) and multi-scale Conv transformation. A representative example of DBB used in our model is shown in Figure 6, which uses equal combination to enhance the conventional Conv. For the 1 × 1 and *K* × *K* branches, the intermediate channel number is set to equal to the input channel number and the initialized 1 × 1 Conv is conducted as the identity matrix. Additionally, other branches are initialized in the conventional way. In addition, a BN layer is added after each convolution to provide nonlinearity during training, which is necessary for performance improvement. We constructed the residual DBB mode, which was embedded in our model.

### 3.2. Squeeze and Excitation Mechanism

**Figure 7:**
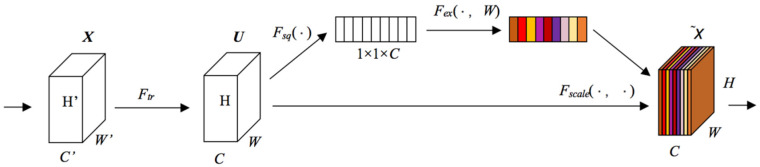
The three steps for SE operation.

Without considering spatial attentions, the repeated Conv operation is not beneficial to extract informative features, and the feature construction method based on a local receptive field is difficult to represent the spatial dependence of the data. Therefore, the attention mechanism SE is adopted [31,32,33,34] to enhance the useful features and to restrain useless ones. By modeling the dynamic non-linearity relationships between the channels, SE can distribute weight to every channel in order to highlight important features and to restrain subordinate features. The SE can be conducted through three steps: “Squeeze” *F_sq_*(·), “Excitation” *F_ex_*(·,*W*), and “Fusion” *F_scale_*(·,*W*), which are shown in Figure 7.

“Squeeze” employs the global average pooling method to perform the feature recalibration. The feature map *X* ∈ *R^H′×W′×C′^* can be transformed into *U* ∈ *R^H×W×C^* after a conventional Conv. The “Squeeze” is based on U, and the input size *H* × *W* × *C* can be condensed into a 1 × 1 × C feature description. The squeeze calculation is performed as per the following equation:

(8) $$z_{c}=F_{sq}\left(u_{c}\right)=\frac{1}{H\times W}\overset{H}{\underset{h=1}{\sum }}\overset{W}{\underset{w=1}{\sum }}u_{c}\left(h,w\right)$$

where *u_c_* represents the c-th feature map; *h*,*w* show the pixel positions.

SE employs “Excitation” to comprehensively capture inter-dependencies between channels. It uses two FC layers to learn the nonlinearity between the channels and to highlight multiple channels instead of just one channel. Given that z represents the global description, σ and *δ* express the Sigmoid and ReLu function, respectively, and *W*_1_ and *W*_2_ represent weight matrix of two FC layers; the “Excitation” is performed as per the following equation:

(9) $$s=F_{ex}\left(z,W\right)=\sigma \left(g\left(z,W\right)\right)=\sigma \left(W_{2}\delta \left(W_{1}z\right)\right)$$

“Fusion” fuses the channel weights s by using the input feature map u, and the formula is shown as

(10) $$\tilde{x_{c}}=F_{scale}\left(u_{c},s_{c}\right)=s_{c}*u_{c}$$

where *s_c_* represents the *c*-th global description.

Due to the superior characteristics of DBB and SE, this paper constructs a multi-scale feature enhancement module which innovatively inserts them into the DS-CapsNet architecture and adds a SE operation after each DBB Conv to obtain the dependence of feature channels, which can increase the learning capability of the model.

### 3.3. Capsules SoftPooling

In the basic capsule network, the primary capsules (Pri-Caps) are directly employed to the structure classification capsules. However, these Pri-Caps, which we called proposal Pri-Caps (ProPri-Caps), only express the fraction of receptive fields and have some redundancy. Through SE operation, important channels have been given more weight than useless channels, which represents the redundancy. Therefore, a new Caps-SoftPool method is improved to obtain Pri-Caps that can reduce the capsule number and can strengthen the representation ability for the detected objects.

**Figure 8:**
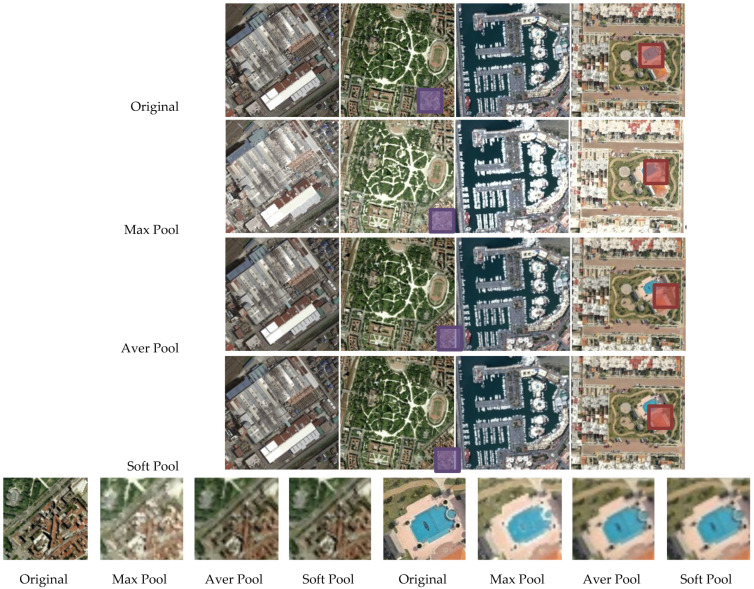
The effects of different pooling methods. The bottom row is the zoom area of the above image.

Maximum pooling or average pooling are two commonly used pooling methods, both of which are speedy and memory efficient but do not retain enough important information in the feature map. Discarding most activations carries the danger of losing important information, and averaging the value of the activations can correspond to local intensity reductions. It has been demonstrated that the SoftPool method can largely preserve descriptive information, while remaining computing- and memory-efficient [35]. The effects of SoftPool are shown in Figure 8, which indicate that this method can better capture detail, while most of the details are lost for max pooling. The zoom area on the bottom row shows that the features are not completely lot such as like maximum selection are not completely lost or restrained by averaging the whole region. The SoftPool method can balance the two methods by using the softmax weighted sum of each part of the region.

The standard pooling method always operates on each element of a region that would confuse the capsule direction in CapsNet. That results in the changing of the attributes of the entity represented by the capsule and leads to learning difficulties. Therefore, different from the standard pooling method, considering that each capsule in CapsNet is a vector, we propose the Caps-SoftPool method to be used between the ProPri-Caps. Caps-SoftPool uses a softmax weighting method to maintain the essential characteristics of the capsule layers while magnifying ProPri-Caps with greater intensity. We define the capsule–response *V_r_* as the activation energy value of a capsule vector, which represents how much the capsule is activated.

(11) $$V_{r}=\sqrt{\sum_{n}^{}{\left(v_{rn}\right)}^{2}}$$

where *v_rn_* is each element value of a capsule vector at the same pixel location in a capsule deck.

The natural exponent (*e*) is adopted to ensure that the larger the energy value of activation, the greater the effect has on the output. Caps-SoftPool employs the smooth maximum approximation for ProPri-Caps. In a capsule deck, all ProPri-Caps within a pooling kernel neighborhood R will be assigned a different proportional coefficient, calculated as

(12) $$w_{i}=\frac{e^{V_{ri}}}{\sum_{j\in R}e^{V_{rj}}}$$

where *i* is the *i*-th ProPri-Caps within a kernel region *R*, *w_i_* is the weight that represents the e ratio of the capsule to the sum of e of all ProPri-Caps. The non-linear transforms imply that larger activation values are more advantageous than smaller values. Notice that each element of a ProPri-Caps shares the same weight, that the output of Caps-SoftPool is a capsule vector, that and each element of a capsule vector is calculated as

(13) $$v_{n}=\underset{i\in R}{\sum }w_{i}*v_{irn}$$

Here, each ProPri-Caps is regarded as a unit, and the normalized result is achieved by the softmax method. The probability distribution is proportional to the value of every ProPri-Caps relative to the adjacent ProPri-Caps within a pooling region. Owing to highlight the activations with greater effect, Caps-SoftPool can show improved recognition performance.

### 3.4. DS-CapsNet Architecture

The main architecture of DS-CapsNet is shown in Figure 4, where several multi-scale feature enhancement modules and the Caps-SoftPool method are inserted into the model. The proposed DS-CapsNet adds four ConvLs with residual-structure DBB and SE and one Caps-SoftPool block.

Four ConvLs were obtained by using a multi-scale feature enhancement module, which employs residual-structure DBB and SE for low-level feature extraction. DBB takes charge of combining different branches with diverse complexities and scales to enrich the feature space in order to generate high-quality feature maps. Additionally, we leveraged SE to enhance the most salient and representative features and to restrain the weaker ones to increase the feature representation capability. We connected DBB and SE in a cascaded way to conduct the augmentation and recalibration for features, respectively.

The next layer is a ProPri-CapsL, which was obtained by reshaping the third convolutional output.

Following the ProPri-CapsL, there is a Pri-CapsL after using the Caps-SoftPool method, which is only performed between the ProPri-Caps is within a pooling region. This layer constructs the Pri-Caps with the strongest responses from the ProPri-Caps, which can reduce the capsule number. In addition, Caps-SoftPool operation further improves the robustness of features and concentrates on the ability of class-specific feature encodings. Since FC CapsL usually takes up most of the parameters of the network, adding CapsPool operation here can significantly lessen the number of parameters.

The last layer is FC CapsL, where the 16D capsule represents one class. The probability that the input vector belongs to a class is represented by the length of the capsule vector, which is represented by ||L2|| in Figure 8. Note that the dynamic routing [36] is processed between this layer and the previous layer.

## 4. Experimental Results and Analysis

In this section, our DS-CapsNet is estimated on the public RS data sets. First, the datasets are presented. Second, the experimental settings are introduced. Third, the performance of experimental results is exhibited.

### 4.1. Datasets Description

**Figure 9:**
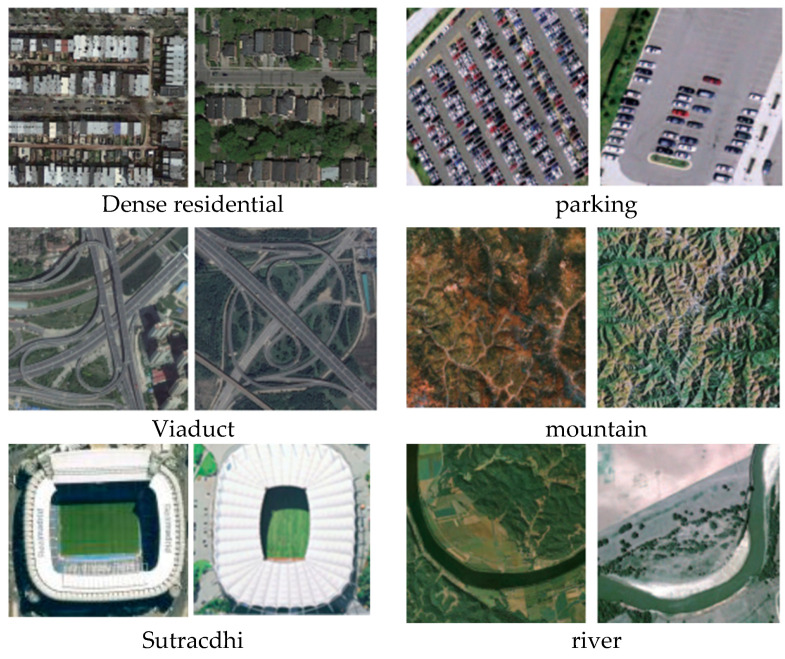
Sample images of the two datasets. First two Columns: AID Database. Second two Columns: NWPU-RESISC45 Dataset.

There were two challenging public datasets that were applied to estimate the proposed DS-CapsNet method. Sample images of the two datasets are displayed in Figure 9. Each data set will then be introduced separately.

AID Data Set: The data set is composed of 10,000 samples (600 × 600 pixels). There are 30 categories in which the number of images is between 220 and 420, and the resolutions of the images are between 0.5 m and 8 m. The images were captured from different RS sensors and platforms during various seasons and under changing weather conditions and under different imaging conditions. The details of this big data set are on its website http://www.captain-whu.com/project/AID/ (15 August 2021).

NWPU-RESISC45 Data Set: The data set contains 31,500 images including 45 categories where each category has 700 images (256 × 256 pixels). The data set was collected from Google Earth, and the resolutions of the images range from 0.2 m to 30 m. The images in each scene category vary greatly in translation, imaging perspective, object pose and appearance, spatial resolution, illumination, background, and occlusion. Some categories have high semantic overlap. The dataset can be obtained on its website http://www.escience.cn/people/JunweiHan/NWPU-RESISC45.html (1 March 2021).

In order to fully estimate the advanced method, two sets of the training–testing ratios were adopted and each data set are randomly divided. For the AID dataset, the training–testing ratios were set as 5:5 and 2:8. For the NWPU-RESISC45 dataset, the ratios were 2:8 and 1:9, respectively.

### 4.2. Experiments Design

In this paper, all of the experiments were conducted on a personal computer using Intel 4.2 GHz 4 core i7-7700 CPU and NVIDIA RTX 2070 GPU with 16-GB memory. The Pytorch framework was adopted to conduct the experiments. All of the images were scaled to 224 × 224 pixels before training. The learning rate was initialized as 0.001 and as plus 0.1 when the loss per epoch did not reduce. For the training process, every *K* × *K* Conv in the DS-CapsNet was replaced by DBB. Additionally, during inference processing, the DBBs were switched into the conventional Conv pattern and were tested. 

The base CapsNet contains three layers: two ConvL and a CapsL. The Convs kernel of the first ConvL is 256, 9 × 9 with 1 stride and ReLU activation function. The second layer is a Pri-CapsL with 32 capsules of an 8-dimensional vector at each pixel position, which was obtained by 8, 9 × 9 Convs kernels with 2 stride based on the output of the proceeding layer. The final layer was a FC-CapsL with a 16D capsule represented as a class.

The parameters employed in the training phase for DS-CapsNet were set by trial and error, as shown in Section 4.3.1. Table 1 describes the detailed parameter settings of the proposed end-to-end training DS-CapsNet.

A total of three evaluation criteria are used: overall accuracy (OA), standard deviation (SD), and confusion matrix (CM). OA is the number of correctly recognized images divided by the total number of test images. CM is a kind of information table where the columns represent the ground-truth and the rows represent the predictions. All of the experiments were operated 10 times, and the OA, SD, and CM of the overall accuracies of the test set are reported.

### 4.3. Results and Analysis

#### 4.3.1. The Analysis about the Parameters Selection

In DS-CapsNet, ConvLs are employed as low-lever feature extractors, and their function is to convert the input images into capsule features. Thus, the ConvL number and the Conv kernel size would influence the recognition performance of the network model. In addition, the Caps-SoftPool kernel size in the capsule pooling process is also an important consideration. The selection and analysis of these parameters are as follows.

**Figure 10:**
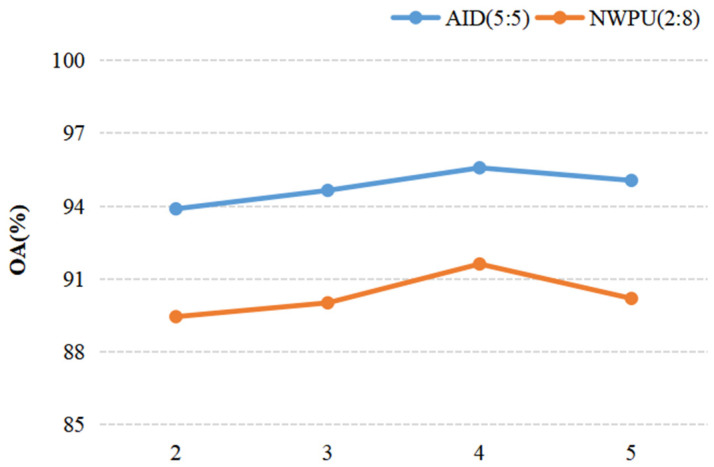
The influence of the ConvLs number.

**Figure 11:**
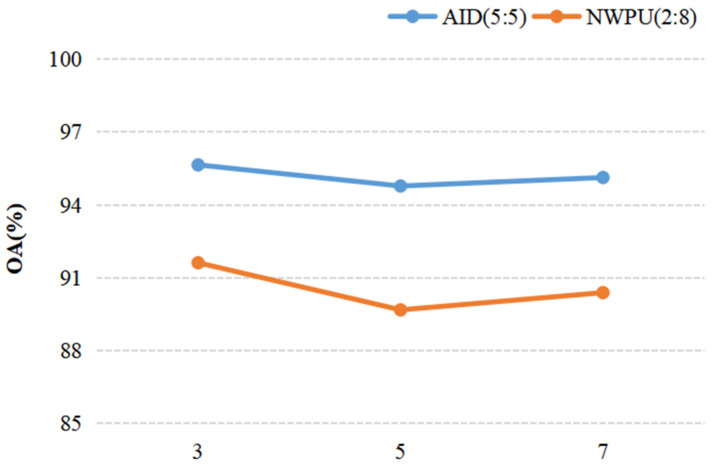
The influence of the Conv kernel size.

**Figure 12:**
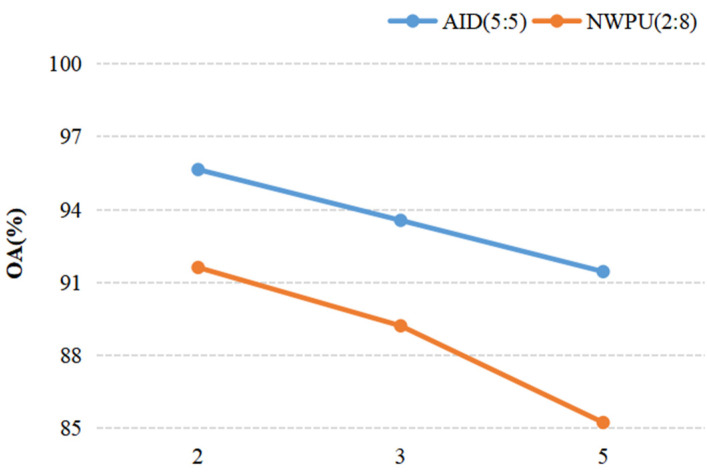
The influence of the pooling kernel size.

When we discussed the effect of a parameter on the result of recognition, other parameters were fixed. For discussing the ConvL number, the kernel size of the Conv and Caps-SoftPool were set to 3 × 3 and 2 × 2, respectively. When the Conv kernel size was analyzed, a 2 × 2 pooling region and 4 ConvLs were used in the DS-CapsNet. Similarly, for analyzing the Caps-SoftPool kernel size, 4 ConvLs and a 3 × 3 Conv kernel were used. Figure 10, Figure 11 and Figure 12 report the different recognition results under different parameter settings. As shown in Figure 10, the recognition effect is better with 4 ConvLs. In addition, 2 and 3 ConvLs models could not extract the features effectively, while the 5 ConvLs model is prone to over fitting. Thus, the ConvLs number was set to 4 in the following experiments. Figure 11 shows the recognition results using different Conv kernel sizes. The performance of the proposed model is better when we used the 3 × 3 Conv kernel size. For the pooling region, the value of 2 obtained the best performance, as shown in Figure 12. Thus, the Caps-SoftPool kernel size 2 × 2 was used in the next experiments.

#### 4.3.2. Performance of DS-CapsNet

In this part, we compared the scene recognition performance of the DS-CapsNet with some state-of-the-art algorithms.

**Figure 13:**
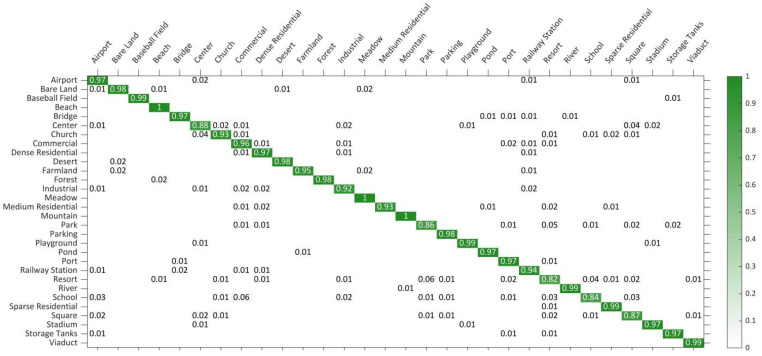
CM on AID dataset under 5:5 training ratio.

The performance test results on the AID dataset: The AID dataset is a large-scale RS scene dataset in which at least 100 images in each scene category are used to train models. All of the comparison algorithms are based on deep convolutional networks. Table 2 shows the comparison results of the different algorithms. As shown, our DS-CapsNet achieves the highest OA of 95.58% ± 0.15% and 93.01% ± 0.16% under the training ratio of 5:5 and 2:8, respectively. In particular, the CNN-CapsNet that combines the VGG_16 and capsule network had an excellent performance on the AID dataset. At the training rate of 5:5 and 2:8, our DS-CapsNet is 0.84% and 1.38% points higher than it, respectively. Furthermore, the recognition OA of the proposed network is 0.34% and is 1.67% higher than that of MF^2^Net, respectively, which is one of the newest RS scene recognition methods. This illuminates that DS-CapsNet can learn more robust feature representation for RS scene images. In Figure 13, the CM of DS-CapsNet is shown to be under the 5:5 training ratio. The data in each row of the CM represents the prediction category of the RS image.

**Figure 14:**
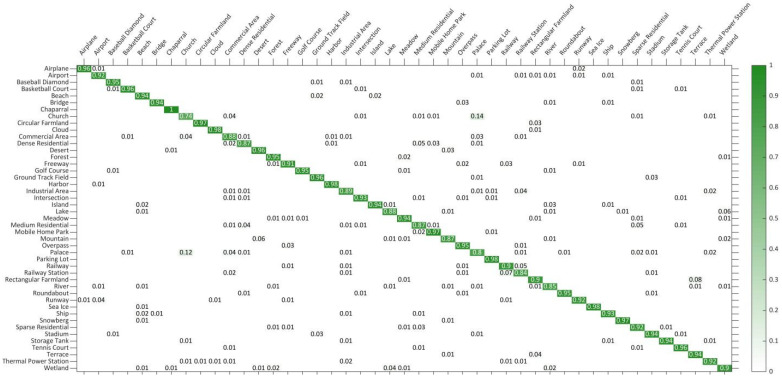
CM on NWPU-RESISC45 dataset under 2:8 training ratio.

Performance test results on the NWPU-RESISC45 dataset: This dataset is a large-scale and one of the most challenging datasets at present. The OA and SD for different methods are shown in Table 3, in which the proposed DS-CapsNet exhibits the best performance. Compared to the second-best model, the enhancements achieved by DS-CapsNet under the 1:9 training ratio are 0.48% (RAN), and those of the 2:8 training ratio are 0.22% (RAN). These excellent results indicate that the proposed method can be competent to accomplish recognition tasks even on the complex and diverse datasets. In addition, the CM of DS-CapsNet is shown in Figure 14 under 2:8 training sets. From CM, it is clearly seen that DS-CapsNet is valid for most categories. The accuracies of DS-CapsNet are higher than 90% in 33 out of 45 classification tasks and are higher than 85% in 42 out of 45 classification tasks. Particularly for the “Chaparral” category, the recognition results for the test images are all correct. These encouraging results prove the availability of the proposed model once more.

#### 4.3.3. Ablation Test

As described in Section 3, our DS-CapsNet includes three main improvements: residual-structure DDB module, SE module, and Caps-SoftPool strategy. To validate their effectiveness on DS-CapsNet, we implemented following ablation experiments:

The experimental results achieved by inserting different modules into the original CapsNet [10] are exhibited in Table 4. “Convs-Caps” expresses the model that substitutes the 2 ConvLs with 9 × 9 conv kernel for 4 ConvLs with 3 × 3 conv kernel. “DBB-CapsNet” expresses the model that uses DBB to replace each conventional 3 × 3 conv. “Convs-SE-Caps” expresses the model that adds the SE module to “Convs-Caps” model, and “DBB-SE-Caps” expresses the model that adopts SE module after each DBB in “DBB-CapsNet” model. The last three models represent how the models adopt different pooling methods in the “DBB-SE-Caps” model.

From the ablation test results, some conclusions can be obtained. At first, among these seven models, “Convs-Caps” has the weakest behavior when using the global features obtained from a simple conv operation. After inserting the DBB and SE modules, the performance of “DBB-SE-Caps” is stronger than that of “Convs-Caps”. Especially, there is an obvious gap between “DBB-SE-Caps” and “Convs-Caps” although it comes at the cost of increasing training parameters. These results display that joint optimization is helpful for the learning of scene representation and that local features are very useful for scene understanding. When the new Caps-SoftPool method is used in the model, “DBB-SE-SoftP-Caps”’s performance is increased for the two challenging datasets, and at the same time, the training parameter number is lessened to a certain extent. This denotes that the Caps-SoftPool method plays an active role in the scene recognition task.

From the experimental results, it can be seen that DBB, SE, and Caps-SoftPool strategies can extract the robust features, which represent a large part of the recognized objects and the structure of the classification capsules using the enhanced Pri-Caps. All of these strategies can contribute to increasing the recognition performance for the original Capsnet.

## 5. Conclusions

In this paper, we have proposed a novel DBB-SE-SoftPool capsule network, known more shortly as DS-CapsNet, in which a new multi-scale feature enhancement module and the new capsule pooling method termed Caps-SoftPool are proposed to improve scene recognition accuracy of RS images by making use of the superiority of CapsNet, the advantages of the residual-structure DBB, the powerful properties of the attention mechanism SE, and the Caps-SoftPool. The DS-CapsNet adopted a deep capsule architecture, which has good application prospects for extracting multiscale capsule features and for restoring high-quality and strong semantic feature representation. By integrating the SE module after each DBB, the proposed DS-CapsNet can improve the feature representation ability and robustness. Moreover, by designing of the Caps-SoftPool module, the proposed DS-CapsNet can largely preserve descriptive information while remaining computing- and memory-efficient. Experimental results from two challenging datasets (AID and NWPU-RESISC45) clearly state that DS-CapsNet reached competitive results compared to a set of existing algorithms. The highest OAs achieved 95.58% and 91.62%, respectively.

## Figures and Tables

**Table 1:** The architecture parameters of DS-CapsNet.

Conv Kernel No.	Size	Stride	Padding	Activation Function
1	3 × 3 × 64	1	No	ReLU
2	3 × 3 × 128	1	No	ReLU
3	3 × 3 × 256	2	No	ReLU
4	3 × 3 × 512	2	No	ReLU
5	2 × 2 × 32	2	No	/
6	8 × 16	1	No	Squash

**Table 2:** OA (%) and SD of different methods under 5:5 and 2:8 training ratios on the AID dataset.

Method	OA (TP5:5)	OA (TP2:8)
GoogLeNet [36]	86.39 ± 0.55	83.44 ± 0.40
VGG-16 [36]	89.64 ± 0.36	86.59 ± 0.29
salM^3^ LBP-CLM [37]	89.76 ± 0.45	86.92 ± 0.35
Fusion by addition [38]	91.87 ± 0.36	/
Fusion by concatenation [38]	91.86 ± 0.28	/
TEX-Net-LF [39]	92.96 ± 0.18	90.87 ± 0.11
Multilever Fusion [40]	94.17 ± 0.32	/
MF^2^Net [41]	94.84 ± 0.27	91.34 ± 0.35
S-CNN [42]	95.24 ± 0.18	92.38 ± 0.13
RAN [43]	93.66 ± 0.28	92.18 ± 0.42
VGG-16-CapsNet [44]	94.74 ± 0.17	91.63 ± 0.19
DS-CapsNet	95.58 ± 0.15	93.01 ± 0.16

**Table 3:** OA (%) and SD of different methods under 2:8 and 1:9 training ratios on the NWPU-RESISC45 dataset.

Method	OA (TP2:8)	OA (TP1:9)
GoogLeNet [45]	78.48 ± 0.26	76.19 ± 0.38
VGG-16 [45]	79.79 ± 0.15	76.47 ± 0.18
Two-Stream Fusion [46]	83.16 ± 0.18	80.22 ± 0.22
BoCF(VGGNet-16) [47]	84.32 ± 0.17	82.65 ± 0.31
TEX-TS-Net [48]	86.36 ± 0.19	84.77 ± 0.24
VGG-VD16+MSCP [49]	88.93 ± 0.14	85.33 ± 0.17
MF^2^Net [41]	89.76 ± 0.27	85.54 ± 0.36
S-CNN [42]	90.99 ± 0.16	88.05 ± 0.78
RAN [43]	91.40 ± 0.30	88.79 ± 0.53
VGG-16-CapsNet [44]	89.18 ± 0.14	85.08 ± 0.13
DS-CapsNet	91.62 ± 0.18	89.27 ± 0.22

**Table 4:** Ablation test results of the proposed DS-CapsNet.

Method	AID Dataset OA (TP5:5)	NWPU-RESISC45 OA (TP2:8)	Prams (M)
Convs-Caps	93.11 ± 0.16	88.85 ± 0.15	8.74
DBB-Caps	93.92 ± 0.15	89.77 ± 0.18	16.04
Convs-SE-Caps	93.85 ± 0.13	90.19 ± 0.14	8.74
DBB-SE-Caps	95.49 ± 0.18	91.56 ± 0.12	16.04
DBB-SE-MaxP	94.28 ± 0.27	90.15 ± 0.16	14.92
DBB-SE-AverP	95.50 ± 0.14	91.53 ± 0.24	14.92
DBB-SE-Caps-SoftP	95.58 ± 0.15	91.62 ± 0.18	14.92

## Data Availability

The websites of the AID and NWPU-RESISC45 datasets are http://www.captain-whu.com/project/AID/; http://www.escience.cn/people/JunweiHan/NWPU-RESISC45.html (accessed on 15 August 2021).
